# Acute Laryngitis—I

**Published:** 1910-01-29

**Authors:** 


### Laryngology and Rhinology.

### ACUTE LARYNGITIS—I.

Acute inflammatory affections of the larynx occur in all degrees of severity, from a mild catarrh to suppurative or even gangrenous inflammation. The really serious inflammations are, however, very rarely seen, while simple acute catarrhal laryngitis is extremely common, and hardly causes serious inconvenience in adults, unless they happen to be professional singers or speakers.

### Acute Catarrhal Laryngitis.

This generally occurs as part of an ordinary "cold," the inflammation reaching the larynx from the nose and naso-pharynx; but it may originate in the larynx, and be caused by the inhalation of dust or chemical or thermal irritants, or by overstrain of the voice. Catarrhal laryngitis is predisposed to by lack of sufficient exercise, by living in overheated and ill-ventilated rooms, by rheumatism, gout and alcoholism, and especially by all forms of nasal obstruction, which lead to the inhalation of unwarmed and unfiltered air through the open mouth. It is also a complication of many of the specific fevers, especially of influenza and measles, and it is common in consumptive patients, quite irrespective of any tuberculous infection of the larynx.

### Signs on Inspection.

The chief alteration in the appearance of the larynx is redness, which is naturally most visible on those parts which are usually pale, namely, the vocal cords and epiglottis. This redness is more or less uniform and diffuse, thus differing from the patchy erythema of secondary syphilis; in the same way both cords are, almost invariably, affected to an equal degree, and marked hyperæmia of one cord is therefore highly suggestive of more serious disease, such as tuberculosis or syphilis. On the epiglottis the mesh-work of vessels is often rendered unduly prominent, and contrasts markedly with the pale yellow of the cartilage which shows through along the edges.

The alteration in colour is, however, most obvious on the vocal cords, and varies from a faint pink to a bright red; in addition, there is sometimes a proliferation and desquamation of the superficial epithelium, and slightly raised white patches may be seen, especially about the centre of each cord, or small erosions may occur on parts subject to attrition, such as the vocal processes and posterior commissure. By reason of the scantiness of the subepithelial tissues much swelling of the cords cannot take place, but there is often a slight tumefaction, sufficient to make the cords come into contact at their centres during phonation, and in this way an acute catarrh may be the starting point of a singer's nodule. There may also be a slight swelling and relaxation of the mucosa in the interarytenoid region, and even a little œdema of the arytenoids; but considerable swelling does not occur in the catarrhal laryngitis of adults; œdematous laryngitis, which is due to more severe forms of infection, will be considered later. There are no large accumulations or strings of mucus, as in chronic laryngitis, but there is usually an increased moisture of the mucosa, and on phonation a little globule of mucus collects and dances on the centre of the vocal cord, and may at first sight be mistaken for a singer's nodule.

### Symptoms.

Of the symptoms, there is generally some local discomfort, dryness of the throat, and irritable cough, but expectoration is scanty unless the catarrh has spread to the trachea and bronchial tubes. A slight tenderness, or rather an increased sensitiveness, may be noticed over the larynx externally, and at the onset there may be malaise and slight feverishness. But the principal symptom is impairment of the voice, which varies in different cases from the slightest hoarseness to complete aphonia. The latter depends on paresis of the adductors, and, though perhaps partly due to inflammatory infiltration of the muscles, is chiefly of the nature of a "functional" aphonia, and bears little relation to the visible degree of inflammation. Thus a hearty man with laryngitis may be expected to speak with complete, though gruff, phonation, while a nervous or anæmic woman will be quite aphonic with the slightest cold.

### Treatment.

Patients rarely apply for treatment when suffering from an ordinary acute catarrhal laryngitis, but if they are professional voice-users prompt treatment becomes of importance. The laryngeal catarrh is often but an extension of a common coryza, which may be aborted or modified in its earliest stage if the patient stay in a warm room, preferably in bed, take a smart aperient, and obtain free diaphoresis with Dover's powder, salicylate of quinine, ammoniated tincture of quinine, or other similar remedy. But this line of treatment is quite useless when the attack has developed, and in such cases, if the larynx is affected, complete rest of the voice must be ordered, alcohol and tobacco forbidden, and sedative steam inhalations, containing benzoic acid, Friar's balsam, or menthol, prescribed.

After the acute stage, or if the patient cannot keep to his room, it is better to use a good atomiser, giving an oily solution of menthol or more stimulating drugs, such as oleum pini sylvestris, eucalyptus, resorcin, or chloretone.

If the nose and naso-pharynx are affected, an alkaline nasal lotion is indicated as well. Internally, expectorants are of value, such as ammonium carbonate, squills and ipecacuanha, and if the cough is severe sedatives may be needed to restrain it, for it greatly increases the congestion of the cords, and for this purpose heroin, gr. $\frac{1}{12}$ to $\frac{1}{6}$, in a lozenge or linctus, is most efficacious.

*(To be continued.)*

